# Onset of effects of non-pharmaceutical interventions on COVID-19 infection rates in 176 countries

**DOI:** 10.1186/s12889-021-11530-0

**Published:** 2021-07-28

**Authors:** Ingo W. Nader, Elisabeth L. Zeilinger, Dana Jomar, Clemens Zauchner

**Affiliations:** 1 IT Power Services GmbH, Modecenterstraße 14/3, A-1030 Vienna, Austria; 2 Faculty of Psychology, University of Vienna, Liebiggasse 5, A-1010 Vienna, Austria

**Keywords:** COVID-19, Coronavirus, Non-pharmaceutical interventions, Mitigation measures, Containment measures, Government measures, Health policy, Machine learning, Accumulated local effect plots, Infection rate, Crosscountry study

## Abstract

**Background:**

During the initial phase of the global COVID-19 outbreak, most countries responded with non-pharmaceutical interventions (NPIs). In this study we investigate the general effectiveness of these NPIs, how long different NPIs need to be in place to take effect, and how long they should be in place for their maximum effect to unfold.

**Methods:**

We used global data and a non-parametric machine learning model to estimate the effects of NPIs in relation to how long they have been in place. We applied a random forest model and used accumulated local effect (ALE) plots to derive estimates of the effectiveness of single NPIs in relation to their implementation date. In addition, we used bootstrap samples to investigate the variability in these ALE plots.

**Results:**

Our results show that closure and regulation of schools was the most important NPI, associated with a pronounced effect about 10 days after implementation. Restrictions of mass gatherings and restrictions and regulations of businesses were found to have a more gradual effect, and social distancing was associated with a delayed effect starting about 18 days after implementation.

**Conclusions:**

Our results can inform political decisions regarding the choice of NPIs and how long they need to be in place to take effect.

**Supplementary Information:**

The online version contains supplementary material available at 10.1186/s12889-021-11530-0.

## Background

Non-pharmaceutical interventions (NPIs) are applied by most countries around the world to reduce the risk of the COVID-19 pandemic and to slow the suspected exponential growth of infections. Exploring the impact of NPIs is crucial for gathering knowledge on effective ways to control the pandemic, and to concurrently avoid unnecessary strain on the general population, both psychologically and economically. The WHO urges that implementation of NPIs during the COVID-19 pandemic must be based on science and evidence [1], and the comparative analysis of the effectiveness of quarantine strategies and contexts was defined as a research gap, that should be addressed with priority [2].

Two rapid reviews on NPIs, one on quarantine measures, one on school closures, concluded that the available evidence was very limited and lacked quality [3, 4]. Recently, there have been some great research efforts to provide comparisons of the general effectiveness of NPIs. Primarily, simulation models have been used to forecast how NPIs will most likely affect infection rates (e.g. [5–7]). However, since multiple aspects of the COVID-19 pandemic are unclear and complex, these simulation methods have to work with parameter assumptions based on fragmented knowledge [3]. Retrospective studies that only consider one country or region at a time in their analyses [8, 9], suffer from the problem that multiple NPIs are introduced simultaneously. Hence, they have no means to distinguish between effects of these interventions and can only evaluate a common effect. One study compared NPIs across 11 European countries [10]. Yet, including only few countries for analysing NPIs means low variability in the data, i.e. implemented NPIs, which can compromise and limit the results. Furthermore, existing studies using Bayesian (e.g. [11]) or econometric methods (e.g. [9]) assume a constant effect of NPIs over time and have no means of estimating the time NPIs need to be effective. Our study wants to address these aspects by applying a different approach.

We used empirical data of 176 countries to evaluate the effectiveness of NPIs on global COVID-19 infection rates with a non-linear machine learning model. This approach allows to estimate the time it takes for each measure to show an effect on infection rates, and how long it takes to reach the maximum effect. Most countries affected by COVID-19 introduced NPIs, but they used different NPIs in different chronological order. This enables our analysis to isolate the effects of single NPIs and provide an estimation for the average effect of each NPI across all countries.

To the best of our knowledge, to date, there are no studies investigating how long it takes for an NPI to take effect, nor comparing NPIs using worldwide data. This, however, could be of great value for policy makers when considering which interventions to apply during a COVID-19 outbreak, when an effect of specific measures is to be expected, and how long it should be in place to reach maximum effect. Our results are a complementary source of information to previous studies on NPIs and may guide decision making on the careful implementation of adequate measures in relation to the COVID-19 pandemic.

## Methods

The aim of our analysis was to examine and compare the effects of NPIs in reducing the initial impact of COVID-19. Our outcome is the daily growth rate, i.e. the relative increase in cumulative confirmed COVID-19 cases from 1 day to the next (growth rate of one indicates no increase, a growth rate of 1.1 a ten-percent increase). Because of the exponential growth during the onset of infectious diseases [12–14], and because we only consider the beginning of the outbreak in our analysis, the growth rate is expected to be constant when no NPIs are in place.

We are interested in understanding the average, world-wide effect of different, isolated NPIs on the growth rate, and, more importantly, how soon these effects start to show after NPI implementation. Most countries have implemented sets of NPIs concurrently, which makes it impossible for within-country-analyses to separate the effect of these NPIs on the growth rate. By focussing on the average effect of NPIs over all countries, our analysis can take advantage of the fact that different countries have implemented different subsets of NPIs. These subsets only show partial overlap, hence allowing the model to disentangle the effects of different NPIs, if there is sufficient variation in the data (see Additional file 1: supplement, section Learning from the data).

We used the CoronaNet [15] dataset and derived features that encode if and how long an NPI has been in place in a specific country at a specific day, and we trained a random forest regression model with the growth rate as the outcome. Additionally, we included two time-dependent but NPI-unrelated covariates (absolute time, i.e. days in relation to 11 March 2020, and time since 25 cumulative COVID-19 cases were reached within each country), and four country-specific covariates. The latter should help to model differences between countries and included the percentage of people being 65 years or older, the percentage of people living in urban territories, the percentage of people that are exposed to air pollution, and the gross domestic product at purchasing power parity (GPD ppp.) per capita (see Additional file 2: extended Methods).

The random forest regression model was chosen because it is a relatively simple and well-established model that can learn non-linear relationships between the features (e.g., NPIs) and the growth rate. We restricted the hyperparameters to avoid overfitting and ensure generalisability of results. To understand how each NPI influenced the growth rate (according to the random forest regression model), accumulated local effect (ALE) plots [16] were used. They show how the growth rate changes in relation to a specific NPI, from 2 weeks prior to implementation to 60 days after. As described above, NPIs were not implemented in isolation, but concurrently with other NPIs in most countries. Even though the subsets of NPIs implemented by different countries showed only partial overlap (allowing the model to separate influences of individual NPIs), the derived features used in the random forest regression model are still correlated. ALE plots are especially suited to handle correlated features [17] (see Additional file 1: supplement, section Learning from the data), aiding to estimate effects of isolated NPIs. To quantify uncertainty and test robustness of results, we additionally re-estimated the model and ALE plots on bootstrap samples (i.e. random resampling of the data; see Additional file 2: extended Methods).

All analyses were carried out using R [18], mainly using the packages mlr3 [19], ranger [20], iml [21], wbstats [22], and ggplot2 [23]. All calculations were performed on an IBM POWER8 platform with 80 threads on 14 physical cores. The code for this analysis was subject to code review among the authors.

## Results

**Fig. 1:**
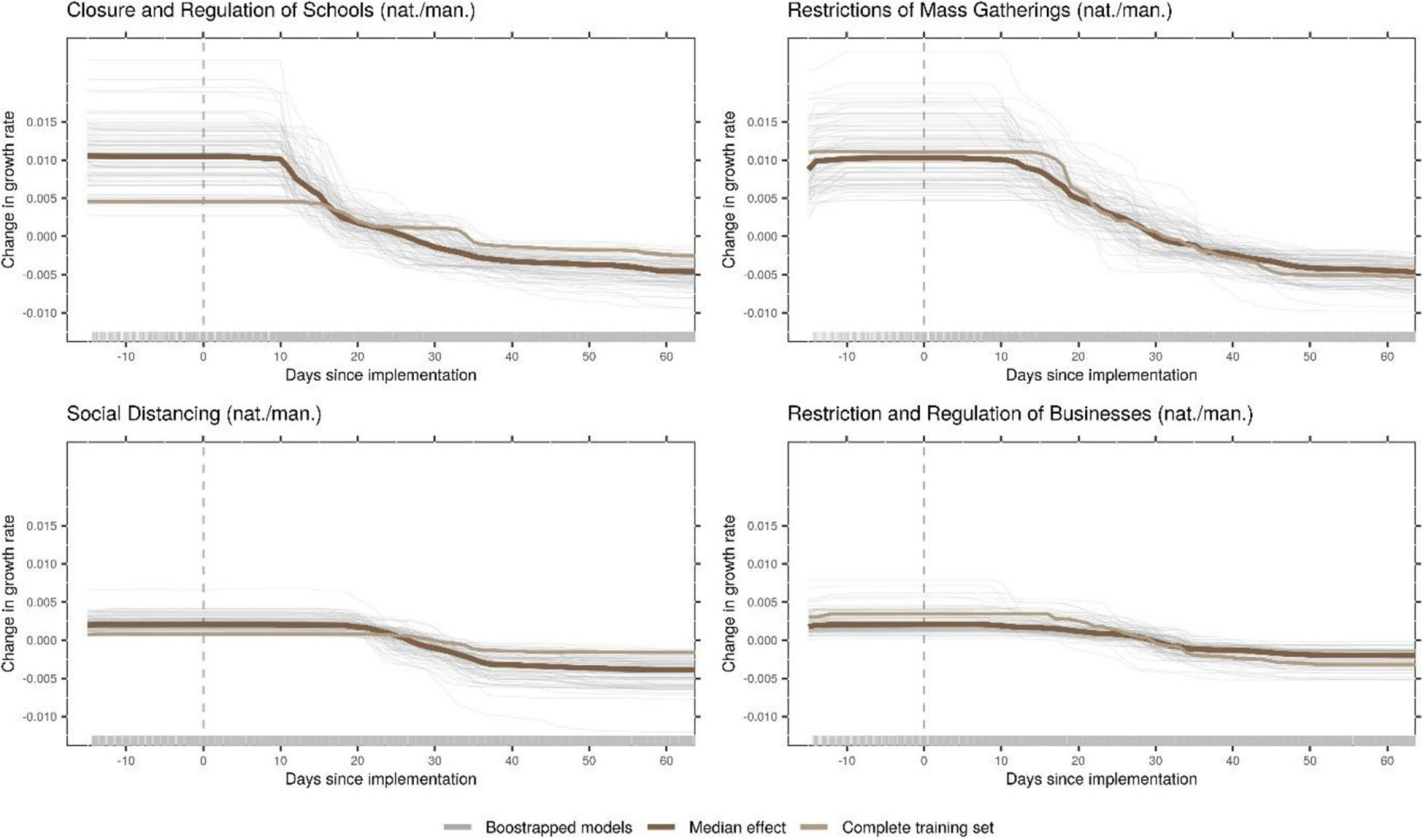
Effects of NPIs as identified by the model. The panels of this figure show predicted changes in the growth rate given how long a certain NPI has been in place. These are the main effects (ALE plots) for the most important NPIs, as identified by the model. The dark brown line is the median effect over all bootstrap samples, grey lines depict individual bootstrap samples, and the light brown line represents the complete training set. The day of implementation is marked with a vertical, dashed line. Plots show a time frame of 2 weeks prior to the measure to 60 days after implementation

The most important NPIs to reduce the growth rate during the COVID-19 outbreak in 2019/2020, as identified by the machine learning model in the context of our data, were closure and regulation of schools, restrictions of mass gatherings, social distancing, and restrictions and regulation of businesses, all with mandatory enforcement on national level (Fig. 1). The first effects started to show about 10 days after implementation, and effects generally lasted until about 40 to 50 days after implementation.

Closure and regulation of schools was associated with a rather distinct drop in the predicted growth rate at around 10 days after implementation of the NPI (Fig. 1). On average over the different countries in the sample, this NPI was introduced relatively timely within each country (see Additional file 1: Supplementary Fig. 1).

Restrictions of Mass Gatherings were associated with a more gradual decrease starting around 10 days after implementation. This NPI was introduced rather early within each country (see Additional file 1: Supplementary Fig. 1). Relatedly, the growth rate was still relatively high, compared to other NPIs (see Additional file 1: Supplementary Table 1).

Social Distancing includes measures like mask wearing, keeping a minimum distance to other individuals, and banning visits to hospitals or other institutions. This NPI was introduced relatively late within each country (see Additional file 1: Supplementary Fig. 1). The reduction of the growth rate started around 18 days after implementation (Fig. 1), with some bootstrap results showing first effects at around 10 days. The maximum decrease was reached 40 days after implementation.

Restriction and Regulation of Businesses started to show slight effects around 10 days after implementation. The total decrease was rather small, compared to other NPIs and to the random variation in the bootstrap samples (Fig. 1).

Further NPIs used in the analysis were associated with only minor effects (see Additional file 1: Supplementary Fig. 2–5 for plots of all NPIs and Supplementary Information for further results and discussion).

**Fig. 2:**
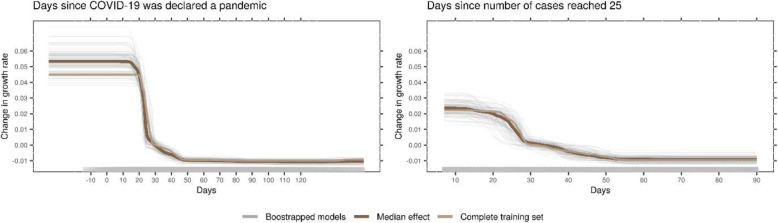
Time-related, NPI-independent effects as identified by the model. The panels show the predicted growth rate given the two time-related, but NPI-independent covariates that were used in the analysis. The panel on the left shows an absolute time scale, indicating the timing in the global COVID-19 outbreak (measured in relation to March 11, 2020, the day the WHO declared COVID-19 a pandemic). The panel on the right is a relative time scale within each country. It indicates how recent an outbreak is within that country (measured in relation to the day that 25 cumulative COVID-19 cases were reached). The dark brown line is the median effect over all bootstrap samples, grey lines depict individual bootstrap samples, and the light brown line represents the complete training set

**Fig. 3:**
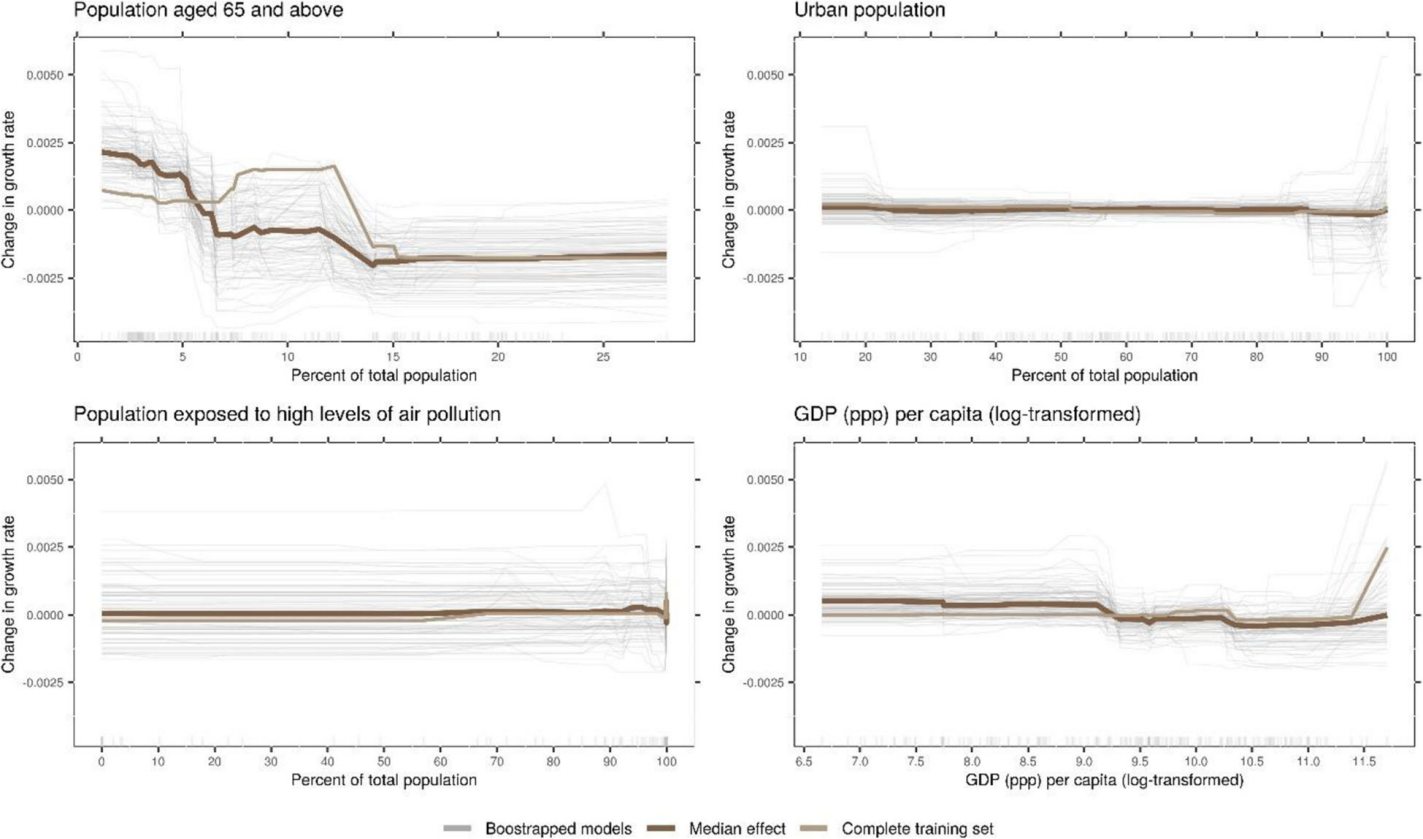
Country-specific covariates and their effects as identified by the model. The panels show the predicted growth rate in relation the four country-specific covariates included in the model. The GDP at purchasing parity power (ppp) per capita was log-transformed to account for the high skewness. The dark brown line is the median effect over all bootstrap samples, grey lines depict individual bootstrap samples, and the light brown line represents the complete training set

Time-related, NPI-independent effects were larger compared to effects associated with NPIs. The average growth rate exhibits a sharp drop around 20 days after the WHO declared COVID-19 a pandemic (Fig. 2). Within countries, the model identified a decrease in growth rate starting from around 2 weeks after 25 cases have been reached, unrelated to NPIs. The relations of country-specific covariates and predicted growth rate were small and hardly exceeded the random variation in the bootstrap samples (Fig. 3; see also Additional file 1: Supplementary Information).

## Discussion

This study investigated the effects of NPIs on the COVID-19 growth rate during the initial phase of the outbreak in 2019/2020. To complement other approaches like simulation studies, we used empirical data to train a non-linear machine learning model and examined the relationship of each NPI with the model’s prediction for the growth rate. We identified four NPIs, all with mandatory enforcement, as most effective: closure and regulation of schools, restrictions of mass gatherings, social distancing, and restrictions and regulation of businesses.

Our approach advances to the current research by investigating how the growth rate changes in the first days and weeks after NPI implementation, providing an estimate of how long it takes for an NPI to take effect. This timing varied between the four most important NPIs, from around 10 to 18 days. Another study that attempted to estimate the onset of effects, which was limited to weekly granularity and a Chinese subpopulation, found similar timing with no effects in the first week, and steadily increasing effects from weeks two to five for the NPIs they considered (emergency declaration, travel ban, and home isolation) [9]. We found that effects of NPIs generally lasted about until 40 to 50 days after implementation. After this time, the maximum possible reduction of the growth rate associated with an NPI has been reached. However, our analysis cannot give an estimate about the possible rise of the growth rate once NPIs are lifted.

Closure and regulation of schools was associated with the most pronounced and earliest drop in the growth rate. The large effect we identified contradicts a rapid review, concluding that school closures might not be effective [4]. However, this conclusion was based on only one simulation study relying on schools data from the UK and transmission dynamics data from China [24]. Our results are supported by a US study [25] that found noticeable effects of school closures, although the methodology did not allow for isolating the effect of school closures from the effect of other concurrently present NPIs. However, it has to be pointed out that school closures have a huge impact on reducing social contacts, as not only the children but also most parents or caregivers need to stay home to supervise their children. This can also lead to closure of small businesses, e.g. if parents are unable to keep their business open while home-schooling their children.

For the other NPIs discussed above, a more gradual drop was observed. A potential reason for this could be a change in intensity of the NPIs during the COVID-19 outbreak. In the beginning, only large gatherings were cancelled, only a few businesses were closed, and social distancing meant maintaining some distance. As the outbreak’s severity increased, so did the NPIs, with more businesses closed, more and smaller gatherings cancelled, and wearing mask becoming increasingly common.

Social distancing showed late effects compared to other NPIs. One possible explanation is that, despite mandatory enforcement, the effectiveness depends on people’s willingness to adhere to this NPI. In face of the perceived negative consequences [26], people might have been reluctant in the beginning, but more willing to follow these rules as the outbreak became increasingly more severe and threatening [27].

Other NPIs used in the analysis were associated with minor effects (see Supplementary Fig. 2–5 for plots of all NPIs). This included lockdown and curfews, which were, on average, used rather late in the outbreak within each country (Supplementary Fig. 1). Despite being associated with minor average effects, some NPIs showed high variation in the bootstrap estimates of their effects. Examples are inbound external border restrictions, voluntary social distancing, and restriction and regulation of government services. Hence, globally, the effects were not significant, but this does not rule out effects in specific countries or under specific circumstances.

There could be multiple reasons for these weak effects. Most importantly, the strategies that are reported in the CoronaNet dataset are not mutually exclusive. For example, restricting mass gatherings reduces the need for travel, which in turn reduces the potential impact of internal and external border restrictions. Additionally, NPIs that have been implemented later, after other NPIs already have been in place, might start from a lower growth rate, hence will have less chance to reduce it. This is especially true if different countries have implemented a similar set of NPIs in similar sequence. The data shows that there is variation in the timing and sequence of NPI implementation across countries (see Supplementary Fig. 1 and Supplementary Notes: Learning from the data; Additional file 1), which at least reduces this concern to some extent.

Also, NPIs that are in effect for a very short timeframe, like curfews or lockdowns that last for only a few days, might have only minor effects which are hard to detect. Similar concerns apply for NPIs on subnational level, which affect fewer people by definition.

In addition to the effects of the NPIs, the model identified strong time-related, but NPI-independent effects, related to the global spread of COVID-19, as well as to the increasing severity within the respective country. An explanation of the global time effect might be that countries affected later could have been better prepared to react to the new disease. The within-country effect might be related to behaviour changes (e.g. hand hygiene) due to the perceived severity of the threat posed by COVID-19 [27], as the number of cases grows and news coverage is high.

When estimating average effects of NPIs on a global scale, it must be acknowledged that different countries are affected differently by COVID-19 [28]. It has been found to follow different patterns in urban and rural areas [29], and spread has been linked to atmospheric pollution [30]. The effects of NPIs have been found to differ by age [31] and relative wealth or poverty [32]. In our analysis, we included country-level covariates as proxies for these differences, but the effects associated with these proxies were only minor. In our opinion, this does not contradict the findings above, but rather shows that between-country differences cannot be modelled effectively by country-level indicators. More fine-grained, individual level data would be necessary, as even something as personal as risk perception [33] and fear [27] can influence the adoption of preventative health behaviours and therefore the effectiveness of NPIs.

A few limitations have to be called out for our study. First, it is an observational study, not an experimental design. Confounding factors, e.g. environmental parameters like climate, which generally influence viral transmission [34] and which vary between countries, cannot be ruled out to influence results. Moreover, the analysis is unable to distinguish between correlation and causation, which makes interpretation of effects difficult. These are limitations that are shared by all observational studies (e.g. [8]).

Another concern is data quality. Confirmed cases are reported daily, but with varying reporting delays [1, 35]. Some countries have changed the case definition for COVID-19 during the outbreak (e.g. China [36]), leading to artificial spikes in the time series of cumulative cases. We tried to mitigate these effects by using a moving average over a full week and by reducing the effect of outliers through winsorisation. Different testing strategies between countries, or systematic underreporting of cases might pose another problem, which we tried to combat by using relative changes within countries (growth rate) as our outcome metric. Furthermore, despite the efforts of the CoronaNet project to standardise NPIs across countries, the reported NPIs are not mutually exclusive, and the individual policies of the countries that are summarised under a certain NPI are sometimes diverse, which might impair the model’s ability to estimate an effect. Even in the face of these data-quality-related problems, the model still explained almost half of variance in the data.

## Conclusions

Despite all limitations mentioned above, we strongly believe that for examining the impact of NPIs on the COVID-19 threat, it is vital to apply diverse methods and different perspectives to gain new insights. In addition to existing empirical studies, our machine-learning-based approach permits to non-parametrically estimate how long it takes for an NPI to affect the growth rate, which has not been studied in detail, yet. Most NPIs started to take effect about 10 days after implementation, like for example closure and regulation of schools, which had the most pronounced effect. One exception was social distancing, which had a delayed effect. We believe that our approach adds knowledge from a different point of view about COVID-19, which may facilitate the evidence-based use of NPIs, as the WHO calls for [1].

## Supplementary Information

**Additional file 1.** Supplementary tables, figures and notes.

**Additional file 2.** Extended Methods.

## Data Availability

All data used in this analysis is publicly available. The time series of cumulative confirmed COVID-19 cases was downloaded from https://data.humdata.org/dataset/novel-coronavirus-2019-ncov-cases, the government response dataset (CoronaNet) was downloaded from https://github.com/saudiwin/corona_tscs, and the Word Bank’s development indicators were downloaded via the R package wbstats [22]. The datasets generated during and/or analysed during the current study as well as the code for the analysis are available from the corresponding author on reasonable request.

## Abbreviations

ALE: Accumulated Local Effect
COVID-19: COrona VIrus Disease 2019
GDP ppp: Gross Domestic Product at purchasing power parity
NPI: Non-Pharmaceutical Intervention
WHO: World Health Organization
