# Supplementary material for: Onset of effects of non-pharmaceutical interventions on COVID-19 infection rates in 176 countries
Source: BMC Public Health. 2021 Jul 28;21:1472. doi: 10.1186/s12889-021-11530-0 (PMC8318058; doi:10.1186/s12889-021-11530-0)
Supplement: Supplementary file 1 — Additional file 1. Supplementary tables, figures and notes. [file 12889_2021_11530_MOESM1_ESM.docx]

**Additional file 2: Supplementary tables, figures, and notes**

**Supplementary Table 1 | NPIs used in the analysis and growth rate at implementation**

| Measure | Level | Enforce-ment | Direction | Countries implemented | Mean growth rate | CI | *n* |
| --- | --- | --- | --- | --- | --- | --- | --- |
| Anti-Disinformation Measures | National | Mandatory |  | 30 | 1.061 | (1.034–1.088) | 18 |
| Closure and Regulation of Schools | National | Mandatory |  | 155 | 1.148 | (1.107–1.190) | 33 |
|  | Sub-national | Mandatory |  | 33 | 1.186 | (1.080–1.302) | 9 |
| Curfew | National | Mandatory |  | 84 | 1.118 | (1.089–1.148) | 33 |
|  | Sub-national | Mandatory |  | 33 | 1.186 | (1.140–1.234) | 19 |
| Declaration of Emergency | National | Mandatory |  | 102 | 1.186 | (1.138–1.235) | 20 |
|  | Sub-national | Mandatory |  | 24 | 1.208 | (1.110–1.315) | 8 |
| External Border Restrictions | National | Mandatory | Outbound | 46 | 1.143 | (1.077–1.214) | 16 |
|  |  |  | Inbound | 131 | 1.180 | (1.125–1.238) | 21 |
|  |  |  | Both | 123 | 1.154 | (1.112–1.197) | 30 |
|  |  |  | (Missing) | 29 | 1.091 | (1.041–1.143) | 15 |
|  |  | Voluntary | Outbound | 43 | 1.151 | (1.080–1.227) | 10 |
| Health Monitoring | National | Mandatory |  | 102 | 1.084 | (1.059–1.109) | 25 |
|  |  | Voluntary |  | 49 | 1.090 | (1.059–1.121) | 17 |
|  | Sub-national | Mandatory |  | 32 | 1.120 | (1.071–1.171) | 10 |
| Health Resources | National | Mandatory |  | 135 | 1.100 | (1.070–1.132) | 39 |
|  |  | Voluntary |  | 118 | 1.126 | (1.097–1.155) | 48 |
|  | Sub-national | Mandatory |  | 44 | 1.158 | (1.101–1.219) | 19 |
|  |  | Voluntary |  | 47 | 1.134 | (1.097–1.172) | 31 |
| Health Testing | National | Mandatory |  | 95 | 1.094 | (1.067–1.121) | 38 |
|  |  | Voluntary |  | 57 | 1.066 | (1.044–1.088) | 36 |
|  | Sub-national | Mandatory |  | 34 | 1.132 | (1.070–1.197) | 18 |
|  |  | Voluntary |  | 22 | 1.130 | (1.078–1.186) | 15 |
| Hygiene | National | Mandatory |  | 57 | 1.056 | (1.031–1.082) | 35 |
|  |  | Voluntary |  | 23 | 1.092 | (1.050–1.137) | 20 |
|  | Sub-national | Mandatory |  | 37 | 1.124 | (1.076–1.173) | 17 |
| Internal Border Restrictions | National | Mandatory |  | 105 | 1.131 | (1.103–1.160) | 46 |
|  | Sub-national | Mandatory |  | 61 | 1.175 | (1.134–1.218) | 33 |
| Lockdown | National | Mandatory |  | 80 | 1.126 | (1.099–1.154) | 50 |
|  | Sub-national | Mandatory |  | 40 | 1.145 | (1.101–1.190) | 25 |
| New Task Force, Bureau or Administrative Configuration | National | Mandatory |  | 106 | 1.130 | (1.089–1.174) | 20 |
|  | Sub-national | Mandatory |  | 31 | 1.142 | (1.082–1.205) | 10 |
| Other Policy Not Listed Above | National | Mandatory |  | 126 | 1.129 | (1.100–1.158) | 46 |
|  |  | Voluntary |  | 98 | 1.098 | (1.068–1.129) | 37 |
|  | Sub-national | Mandatory |  | 41 | 1.137 | (1.093–1.183) | 23 |
|  |  | Voluntary |  | 29 | 1.191 | (1.137–1.247) | 18 |
| Public Awareness Measures | National | Mandatory |  | 65 | 1.124 | (1.082–1.166) | 21 |
|  |  | Voluntary |  | 127 | 1.140 | (1.105–1.176) | 32 |
|  | Sub-national | Mandatory |  | 28 | 1.099 | (1.032–1.170) | 11 |
|  |  | Voluntary |  | 34 | 1.119 | (1.041–1.203) | 10 |
| Quarantine | National | Mandatory |  | 147 | 1.108 | (1.070–1.147) | 28 |
|  |  | Voluntary |  | 38 | 1.153 | (1.079–1.231) | 9 |
|  | Sub-national | Mandatory |  | 41 | 1.140 | (1.088–1.195) | 16 |
| Restriction and Regulation of Businesses | National | Mandatory |  | 138 | 1.146 | (1.113–1.181) | 48 |
|  |  | Voluntary |  | 34 | 1.112 | (1.066–1.159) | 21 |
|  | Sub-national | Mandatory |  | 63 | 1.165 | (1.127–1.205) | 33 |
|  |  | Voluntary |  | 21 | 1.156 | (1.088–1.229) | 13 |
| Restriction and Regulation of Government Services | National | Mandatory |  | 101 | 1.149 | (1.114–1.185) | 36 |
|  |  | Voluntary |  | 23 | 1.145 | (1.059–1.238) | 8 |
|  | Sub-national | Mandatory |  | 32 | 1.167 | (1.110–1.228) | 17 |
| Restrictions of Mass Gatherings | National | Mandatory |  | 152 | 1.177 | (1.132–1.224) | 25 |
|  |  | Voluntary |  | 50 | 1.098 | (1.048–1.150) | 16 |
|  | Sub-national | Mandatory |  | 51 | 1.156 | (1.106–1.208) | 22 |
| Social Distancing | National | Mandatory |  | 115 | 1.134 | (1.110–1.160) | 63 |
|  |  | Voluntary |  | 63 | 1.141 | (1.097–1.185) | 26 |
|  | Sub-national | Mandatory |  | 41 | 1.159 | (1.119–1.202) | 31 |

The table shows the number of countries that an NPI was implemented in, the (geometric) mean of the growth rate on the day that the NPI was implemented, together with a 95% confidence interval (CI) and the sample size *n* for estimating this mean and CI. Note that this sample size is often considerably smaller than the number of countries that have implemented that NPI, as data with less than 25 cases was excluded from analysis.

**Supplementary Table 2 | Countries included in the analysis**

| Country | *n* | Dataset | RMSE | R² |
| --- | --- | --- | --- | --- |
| Afghanistan (AFG) | 84 | test | 0.0253 | 0.522 |
| Albania (ALB) | 84 | training | 0.0210 | 0.718 |
| Algeria (DZA) | 84 | training | 0.0277 | 0.822 |
| Andorra (AND) | 84 | training | 0.0387 | 0.559 |
| Angola (AGO) | 84 | test | 0.0231 | -1.217 |
| Antigua and Barbuda (ATG) | 84 | training | 0.0508 | -0.648 |
| Argentina (ARG) | 84 | test | 0.0355 | 0.733 |
| Armenia (ARM) | 84 | training | 0.0331 | 0.527 |
| Australia (AUS) | 84 | training | 0.0414 | 0.788 |
| Austria (AUT) | 84 | training | 0.0629 | 0.677 |
| Azerbaijan (AZE) | 84 | training | 0.0263 | 0.784 |
| Bahamas (BHS) | 84 | training | 0.0274 | -1.120 |
| Bahrain (BHR) | 84 | training | 0.0380 | -0.191 |
| Bangladesh (BGD) | 84 | test | 0.0842 | -0.105 |
| Barbados (BRB) | 84 | test | 0.0378 | -1.952 |
| Belarus (BLR) | 84 | test | 0.0773 | 0.046 |
| Belgium (BEL) | 84 | training | 0.0492 | 0.682 |
| Belize (BLZ) | 41 | training | 0.0452 | 0.455 |
| Benin (BEN) | 84 | training | 0.0520 | -0.261 |
| Bhutan (BTN) | 78 | training | 0.0221 | -0.520 |
| Bolivia (BOL) | 84 | training | 0.0281 | 0.219 |
| Bosnia and Herzegovina (BIH) | 84 | test | 0.0394 | 0.605 |
| Botswana (BWA) | 84 | test | 0.0424 | -0.233 |
| Brazil (BRA) | 84 | training | 0.0611 | 0.505 |
| Brunei (BRN) | 84 | test | 0.0555 | -3.513 |
| Bulgaria (BGR) | 84 | training | 0.0259 | 0.693 |
| Burkina Faso (BFA) | 84 | training | 0.0341 | 0.516 |
| Burma (MMR) | 84 | training | 0.0302 | 0.151 |
| Burundi (BDI) | 84 | test | 0.0276 | -0.461 |
| Cabo Verde (CPV) | 84 | test | 0.0201 | -0.132 |
| Cambodia (KHM) | 84 | test | 0.0538 | -1.953 |
| Cameroon (CMR) | 84 | training | 0.0410 | 0.589 |
| Canada (CAN) | 84 | test | 0.0555 | 0.658 |
| Central African Republic (CAF) | 84 | training | 0.0296 | 0.465 |
| Chad (TCD) | 84 | training | 0.0381 | 0.513 |
| Chile (CHL) | 84 | training | 0.0538 | 0.555 |
| China (CHN) | 78 | test | 0.0813 | -0.984 |
| Colombia (COL) | 84 | test | 0.0296 | 0.741 |
| Comoros (COM) | 83 | training | 0.0285 | -0.237 |
| Congo (Brazzaville) (COG) | 84 | test | 0.0274 | 0.272 |
| Congo (Kinshasa) (COD) | 84 | training | 0.0199 | 0.469 |
| Costa Rica (CRI) | 84 | training | 0.0321 | 0.718 |
| Cote d'Ivoire (CIV) | 84 | test | 0.0231 | 0.701 |
| Croatia (HRV) | 84 | training | 0.0370 | 0.781 |
| Cuba (CUB) | 84 | training | 0.0209 | 0.872 |
| Cyprus (CYP) | 84 | test | 0.0246 | 0.797 |
| Czechia (CZE) | 84 | training | 0.0473 | 0.740 |
| Denmark (DNK) | 84 | training | 0.0455 | 0.401 |
| Djibouti (DJI) | 84 | test | 0.0543 | 0.409 |
| Dominican Republic (DOM) | 84 | training | 0.0355 | 0.722 |
| Ecuador (ECU) | 84 | test | 0.0588 | 0.555 |
| Egypt (EGY) | 84 | test | 0.0232 | 0.641 |
| El Salvador (SLV) | 84 | test | 0.0241 | 0.333 |
| Equatorial Guinea (GNQ) | 84 | test | 0.0416 | 0.302 |
| Eritrea (ERI) | 84 | training | 0.0592 | -0.613 |
| Estonia (EST) | 84 | training | 0.0318 | 0.383 |
| Eswatini (SWZ) | 84 | training | 0.0272 | 0.251 |
| Ethiopia (ETH) | 84 | training | 0.0347 | -0.559 |
| Fiji (FJI) | 33 | training | 0.0547 | -455.624 |
| Finland (FIN) | 84 | test | 0.0410 | 0.675 |
| France (FRA) | 84 | training | 0.0595 | 0.691 |
| Gabon (GAB) | 84 | training | 0.0337 | 0.243 |
| Gambia (GMB) | 81 | training | 0.0582 | -0.380 |
| Georgia (GEO) | 84 | test | 0.0325 | -0.002 |
| Germany (DEU) | 84 | training | 0.0727 | 0.635 |
| Ghana (GHA) | 84 | training | 0.0322 | 0.432 |
| Greece (GRC) | 84 | test | 0.0518 | 0.438 |
| Guatemala (GTM) | 84 | training | 0.0323 | -0.624 |
| Guinea (GIN) | 84 | training | 0.0286 | 0.679 |
| Guinea-Bissau (GNB) | 84 | test | 0.0659 | 0.135 |
| Guyana (GUY) | 84 | training | 0.0282 | -2.695 |
| Haiti (HTI) | 84 | training | 0.0480 | -0.329 |
| Honduras (HND) | 84 | training | 0.0375 | 0.491 |
| Hungary (HUN) | 84 | test | 0.0223 | 0.876 |
| Iceland (ISL) | 84 | training | 0.0282 | 0.825 |
| India (IND) | 84 | training | 0.0413 | 0.454 |
| Indonesia (IDN) | 84 | training | 0.0434 | 0.648 |
| Iran (IRN) | 84 | test | 0.0658 | 0.555 |
| Iraq (IRQ) | 84 | training | 0.0177 | 0.801 |
| Ireland (IRL) | 84 | training | 0.0525 | 0.693 |
| Israel (ISR) | 84 | training | 0.0529 | 0.699 |
| Italy (ITA) | 84 | test | 0.0568 | 0.676 |
| Jamaica (JAM) | 84 | training | 0.0363 | 0.313 |
| Japan (JPN) | 84 | training | 0.0403 | -0.008 |
| Jordan (JOR) | 84 | training | 0.0355 | 0.355 |
| Kazakhstan (KAZ) | 84 | training | 0.0357 | 0.623 |
| Kenya (KEN) | 84 | training | 0.0297 | 0.481 |
| Korea, South (KOR) | 84 | test | 0.0948 | 0.299 |
| Kuwait (KWT) | 84 | training | 0.0589 | -3.880 |
| Kyrgyzstan (KGZ) | 84 | training | 0.0167 | 0.814 |
| Latvia (LVA) | 84 | training | 0.0304 | 0.751 |
| Lebanon (LBN) | 84 | test | 0.0345 | 0.488 |
| Lesotho (LSO) | 44 | test | 0.0701 | -0.026 |
| Liberia (LBR) | 84 | training | 0.0268 | -0.668 |
| Libya (LBY) | 84 | training | 0.0397 | -0.304 |
| Liechtenstein (LIE) | 84 | test | 0.0462 | -3.164 |
| Lithuania (LTU) | 84 | test | 0.0548 | 0.516 |
| Luxembourg (LUX) | 84 | training | 0.0563 | 0.609 |
| Madagascar (MDG) | 84 | test | 0.0347 | -0.389 |
| Malawi (MWI) | 84 | test | 0.0501 | -0.323 |
| Malaysia (MYS) | 84 | test | 0.0544 | 0.554 |
| Maldives (MDV) | 84 | test | 0.0527 | 0.361 |
| Mali (MLI) | 84 | training | 0.0190 | 0.759 |
| Malta (MLT) | 84 | test | 0.0429 | -0.021 |
| Mauritania (MRT) | 84 | training | 0.0524 | 0.256 |
| Mauritius (MUS) | 84 | test | 0.0438 | 0.224 |
| Mexico (MEX) | 84 | test | 0.0403 | 0.607 |
| Moldova (MDA) | 84 | training | 0.0273 | 0.800 |
| Monaco (MCO) | 84 | training | 0.0408 | -2.448 |
| Mongolia (MNG) | 84 | training | 0.0501 | -0.208 |
| Montenegro (MNE) | 84 | test | 0.0356 | 0.317 |
| Morocco (MAR) | 84 | test | 0.0345 | 0.762 |
| Mozambique (MOZ) | 84 | test | 0.0264 | -0.288 |
| Namibia (NAM) | 71 | training | 0.0585 | -0.452 |
| Nepal (NPL) | 84 | test | 0.0478 | -0.247 |
| Netherlands (NLD) | 84 | training | 0.0437 | 0.755 |
| New Zealand (NZL) | 84 | test | 0.0475 | 0.661 |
| Nicaragua (NIC) | 84 | training | 0.0806 | 0.226 |
| Niger (NER) | 84 | test | 0.0549 | 0.359 |
| Nigeria (NGA) | 84 | training | 0.0295 | 0.510 |
| North Macedonia (MKD) | 84 | test | 0.0245 | 0.797 |
| Norway (NOR) | 84 | training | 0.0608 | 0.602 |
| Oman (OMN) | 84 | training | 0.0234 | 0.511 |
| Pakistan (PAK) | 84 | test | 0.0438 | 0.538 |
| Panama (PAN) | 84 | training | 0.0383 | 0.760 |
| Paraguay (PRY) | 84 | training | 0.0256 | 0.307 |
| Peru (PER) | 84 | training | 0.0596 | 0.317 |
| Philippines (PHL) | 84 | training | 0.0300 | 0.824 |
| Poland (POL) | 84 | training | 0.0381 | 0.773 |
| Portugal (PRT) | 84 | training | 0.0609 | 0.688 |
| Qatar (QAT) | 84 | training | 0.0521 | -1.631 |
| Romania (ROU) | 84 | test | 0.0340 | 0.818 |
| Russia (RUS) | 84 | test | 0.0759 | 0.164 |
| Rwanda (RWA) | 84 | training | 0.0315 | -1.221 |
| Saint Vincent and the Grenadines (VCT) | 75 | training | 0.0521 | -10.031 |
| San Marino (SMR) | 84 | training | 0.0356 | -0.080 |
| Sao Tome and Principe (STP) | 84 | training | 0.0291 | -0.482 |
| Saudi Arabia (SAU) | 84 | training | 0.0420 | 0.538 |
| Senegal (SEN) | 84 | training | 0.0301 | 0.422 |
| Serbia (SRB) | 84 | training | 0.0327 | 0.816 |
| Seychelles (SYC) | 44 | training | 0.0565 | -35.457 |
| Sierra Leone (SLE) | 84 | test | 0.0256 | 0.581 |
| Singapore (SGP) | 84 | training | 0.0647 | -2.003 |
| Slovakia (SVK) | 84 | training | 0.0277 | 0.708 |
| Slovenia (SVN) | 84 | training | 0.0401 | 0.481 |
| Somalia (SOM) | 84 | training | 0.0453 | 0.434 |
| South Africa (ZAF) | 84 | test | 0.0543 | 0.465 |
| South Sudan (SSD) | 84 | training | 0.0497 | 0.305 |
| Spain (ESP) | 84 | training | 0.0729 | 0.640 |
| Sri Lanka (LKA) | 84 | test | 0.0437 | -0.982 |
| Sudan (SDN) | 84 | training | 0.0500 | 0.420 |
| Suriname (SUR) | 71 | test | 0.0249 | 0.085 |
| Sweden (SWE) | 84 | training | 0.0432 | 0.645 |
| Switzerland (CHE) | 84 | training | 0.0592 | 0.702 |
| Syria (SYR) | 84 | test | 0.0488 | -2.579 |
| Taiwan* (TWN) | 84 | test | 0.0618 | -0.909 |
| Tajikistan (TJK) | 84 | test | 0.0479 | 0.369 |
| Tanzania (TZA) | 84 | training | 0.0573 | 0.364 |
| Thailand (THA) | 84 | test | 0.0904 | -0.462 |
| Togo (TGO) | 84 | test | 0.0319 | -0.209 |
| Trinidad and Tobago (TTO) | 84 | test | 0.0518 | -7.081 |
| Tunisia (TUN) | 84 | test | 0.0390 | 0.661 |
| Turkey (TUR) | 84 | test | 0.0646 | 0.607 |
| US (USA) | 84 | training | 0.0938 | 0.450 |
| Uganda (UGA) | 84 | training | 0.0465 | -0.955 |
| Ukraine (UKR) | 84 | test | 0.0523 | 0.590 |
| United Arab Emirates (ARE) | 84 | training | 0.0440 | 0.367 |
| United Kingdom (GBR) | 84 | test | 0.0601 | 0.653 |
| Uruguay (URY) | 84 | test | 0.0380 | 0.356 |
| Uzbekistan (UZB) | 84 | training | 0.0321 | 0.697 |
| Venezuela (VEN) | 84 | test | 0.0442 | -0.829 |
| Vietnam (VNM) | 84 | training | 0.0461 | -0.546 |
| West Bank and Gaza (PSE) | 84 | training | 0.0409 | -0.151 |
| Yemen (YEM) | 84 | test | 0.0359 | 0.387 |
| Zambia (ZMB) | 84 | training | 0.0549 | -0.283 |
| Zimbabwe (ZWE) | 84 | test | 0.0506 | -0.334 |

The table shows the number of days used in the analysis for each country, the dataset the country was part of (training set or test set), the root mean squared error (RMSE) and the explained variance (R^2^) of the predicted growth rate for that specific country.

**Supplementary Table 3 | Fit statistics**

|  | *n* | Countries | RMSE | Bootstrap RMSE | R^2^ | Bootstrap R^2^ |
| --- | --- | --- | --- | --- | --- | --- |
| Training set | 8738 | 106 | 0.044 | 0.0412 (0.0005) | 0.558 | 0.607 (0.010) |
| Test set | 5821 | 70 | 0.049 | 0.0475 (0.0005) | 0.442 | 0.471 (0.012) |

The table shows the sample size *n*, the number of countries in the sample, root mean squared error (RMSE) and explained variance (*R*^2^) of the model estimated on the complete training set, as well as mean RMSE and *R*^2^ (with their standard deviation) of the bootstrapped models in the training and test set.


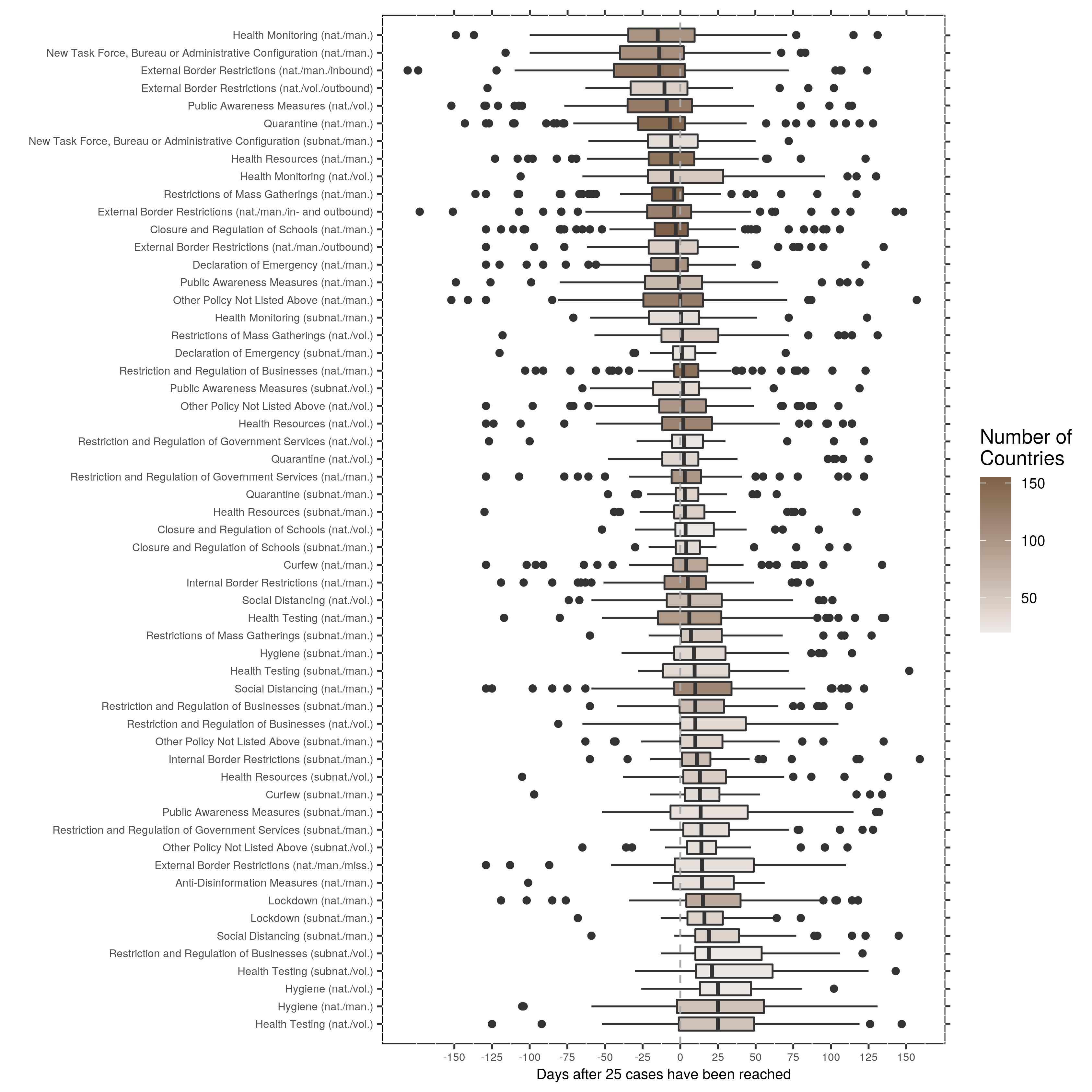


**Supplementary Fig. 1 | Time point of NPI implementation.** The figure shows the time point at which a certain NPI was implemented, in relation to the day when a cumulative number of 25 confirmed COVID-19 cases was reached. Each data point represents one country, and the boxplots show the distribution of when these countries implemented the NPI. Colour coding corresponds to the number of countries that have implemented a specific NPI.





**Supplementary Fig. 2 | Effects of all NPIs as identified by the model (part 1).** The panels of this figure show predicted changes in the growth rate given how long a certain NPI has been in place. These are the main effects (ALE plots) for the most important NPIs, as identified by the model. The dark brown line is the median effect over all bootstrap samples, grey lines depict individual bootstrap samples, and the light brown line represents the complete training set. The day of implementation is marked with a vertical, dashed line. Plots show a time frame of two weeks prior to the measure to 60 days after implementation.





**Supplementary Fig. 3 | Effects of all NPIs as identified by the model (part 2).** The panels of this figure show predicted changes in the growth rate given how long a certain NPI has been in place. These are the main effects (ALE plots) for the most important NPIs, as identified by the model. The dark brown line is the median effect over all bootstrap samples, grey lines depict individual bootstrap samples, and the light brown line represents the complete training set. The day of implementation is marked with a vertical, dashed line. Plots show a time frame of two weeks prior to the measure to 60 days after implementation.





**Supplementary Fig. 4 | Effects of all NPIs as identified by the model (part 3).** The panels of this figure show predicted changes in the growth rate given how long a certain NPI has been in place. These are the main effects (ALE plots) for the most important NPIs, as identified by the model. The dark brown line is the median effect over all bootstrap samples, grey lines depict individual bootstrap samples, and the light brown line represents the complete training set. The day of implementation is marked with a vertical, dashed line. Plots show a time frame of two weeks prior to the measure to 60 days after implementation.





**Supplementary Fig. 5 | Effects of all NPIs as identified by the model (part 4).** The panels of this figure show predicted changes in the growth rate given how long a certain NPI has been in place. These are the main effects (ALE plots) for the most important NPIs, as identified by the model. The dark brown line is the median effect over all bootstrap samples, grey lines depict individual bootstrap samples, and the light brown line represents the complete training set. The day of implementation is marked with a vertical, dashed line. Plots show a time frame of two weeks prior to the measure to 60 days after implementation.


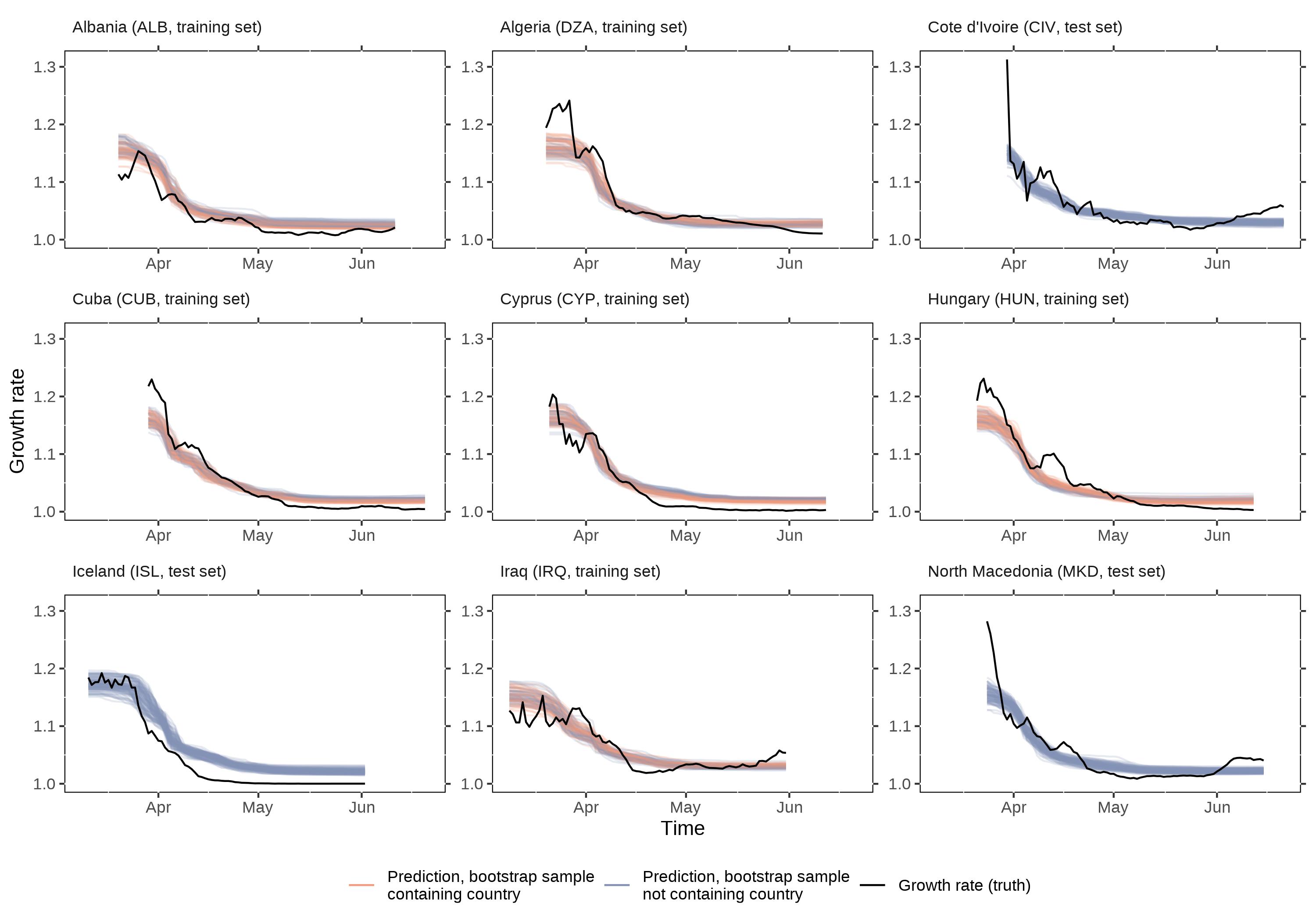


**Supplementary Fig. 6 | Country-specific growth rates and well-approximating predictions.** The figure shows some examples of countries that the models’ predictions were reasonably accurate. The models were only able to learn average effects of the NPIs over all countries, hence they were not able to predict untypically high (e.g., DZA, CIV, MKD) or low (e.g., ALB) growth rates in the beginning of the outbreak. They were also not capable to predict rising growth rates, neither in the mid-sections of the time series (e.g., HUN, MKD) nor towards the end (e.g., CIV, IRQ, MKD). Another observation that can be made is that the models were not always able to learn the full extent in the reduction of the growth rate, as the predicted growth rates are higher than the actual growth rates towards the end of the time series (e.g., DZA, CYP, ISL). Countries that were part of the training set contain bootstrap estimates of the predictions where the respective country was part of the bootstrap sample or not (indicated by the colour of the bootstrap estimates), countries that were in the test set contain only bootstrap estimates that did not contain the respective country.


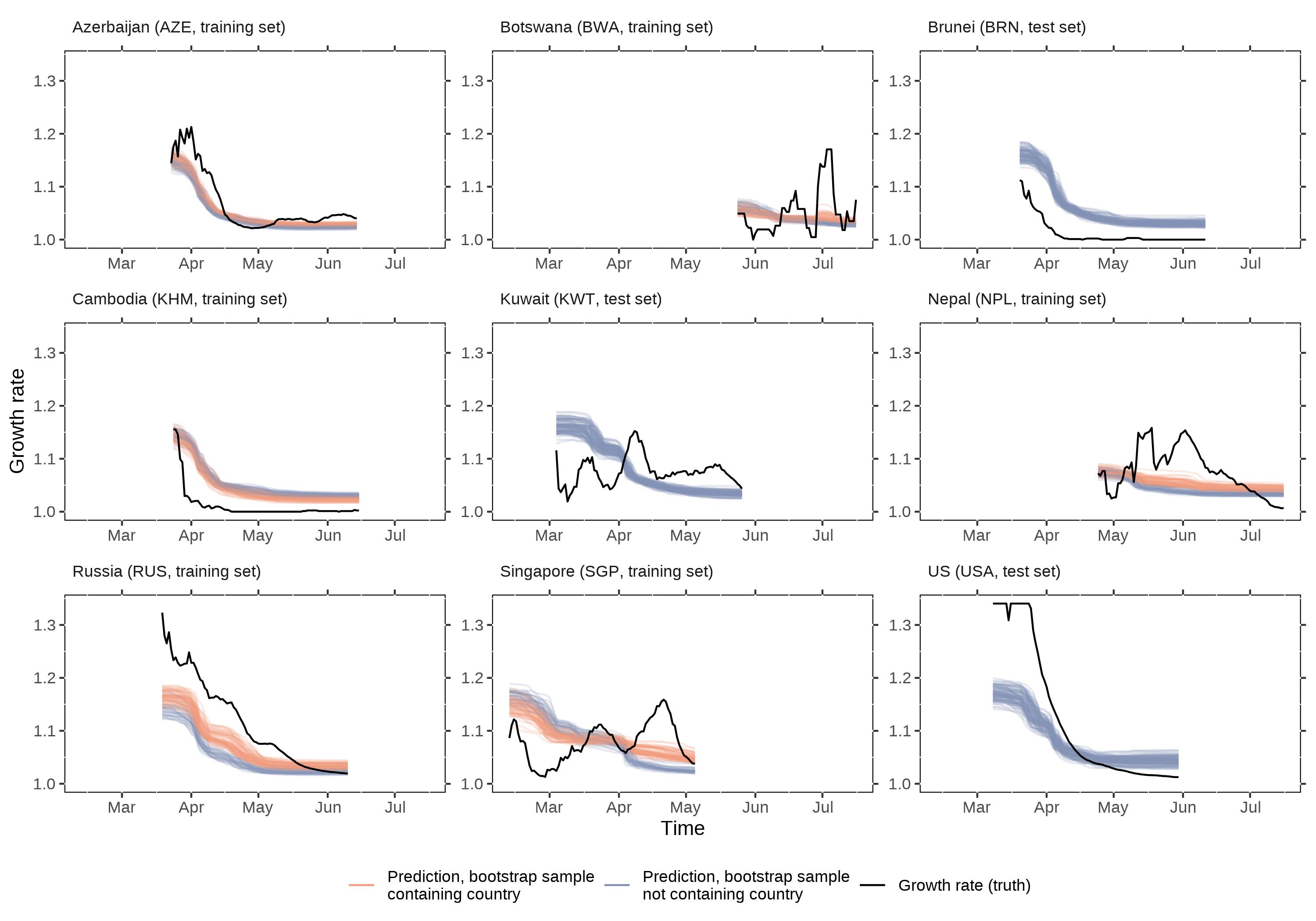


**Supplementary Fig. 7 | Country-specific growth rates and inaccurate predictions.** The figure shows some examples of countries that the models’ predictions were inaccurate. For some countries, the predicted growth rate was systematically higher (e.g., BRN, KHM) or lower (e.g., RUS) than the actual growth rate. In some instances, the reduction in the growth rate was predicted too early (e.g., AZE) or too late (e.g., KHM, BRN). The models were not able to predict very high growth rates in the beginning of the outbreak (e.g., USA, RUS), nor were they able to capture time series with untypical trends in the growth rate (e.g., BWA, KWT, NPL, SGP). Countries that were part of the training set contain bootstrap estimates of the predictions where the respective country was part of the bootstrap sample or not (indicated by the colour of the bootstrap estimates), countries that were in the test set contain only bootstrap estimates that did not contain the respective country.

**Supplementary Notes**

Learning from the data

In contrast to analysing the cumulative confirmed COVID-19 cases for individual countries as individual time series, the approach used in this study aims at estimating the mean effect of a non-pharmaceutical intervention (NPI) over all countries that have implemented it. The effect is quantified by how much the current growth rate at a specific point in time (relative to the implementation date) differs between countries that have implemented the measure vs. countries that have not implemented it, as well as how it differs at specific times after implementation (i.e., the growth rate is expected to be influenced differently by an NPI on the first day after implementation compared to the second or third day, etc.). Assuming the growth rate to be constant during the initial phase of exponential growth with no NPIs in place, a lower-than-normal growth rate should co-occur with an NPI being in place, or, more realistically, a set of NPIs being in place at the same time. This assumption is expected to be valid both for the time domain, as well as geographically. It should not matter in which country these measures are in place, as we are only interested in an average effect of these NPIs. This allows the machine learning model to disentangle the effects of different NPIs, as different countries have implemented different subsets of all possible NPIs,

However, NPIs that have been implemented later, after other NPIs already have been in place, might start from a lower growth rate, hence will have less chance to reduce it. This concern is more severe if different countries have implemented a similar set of NPIs in similar sequence, and less severe if they have done so in different sequence with different timing during the spread of the COVID-19 pandemic, which will be investigated in the following paragraphs.

Within each country, the features representing the NPIs are highly correlated (all Pearson correlations *r* > .69, with a median correlation of *r* = .96), because a country has either not implemented a pair of measures (resulting in a correlation of one), or it has implemented two measures in close succession, leading to a very high correlation.

However, in the model we do not distinguish between different countries. We want to derive the average effect over all countries. We assume that the dependent variable in the model, the growth rate, covaries with an NPI being in place irrespective of which country that data point has originated from. Hence, as long as different countries have implemented sufficiently different subsets of NPIs, the model can estimate the effect of an NPI. Different countries having implemented different subsets of NPIs would result in lower correlations of the NPI-related features across all countries.

Another characteristic that can help the model learn the effects of different NPIs is the chronological order that different countries have implemented different NPIs. Different chronological order in different countries would also result in lower correlations of the measures across all countries. And the correlations are indeed lower when calculated across (and not within) countries (minimum Pearson correlation *r* = -0.42, median correlation of *r* = 0.14, maximum at *r* = 0.72), substantiating the assumption that different countries have implemented different subsets and/or have implemented the NPIs in different chronological order.

Additional discussion of results: Country-specific covariates

The relations of country-specific covariates and predicted growth rate were relatively small compared to NPIs and time-related, NPI-independent effects, and the random variation in the bootstrap samples was large (Fig. 3). The largest effect in this group of covariates was the percentage of the population aged 65 and above. Countries with a lower percentage, i.e., countries with a younger population, showed slightly higher growth rates. The effect was marginally larger than the bootstrap variation.

Countries with high low GDP (ppp) per capita seemed to have slightly higher growth rates on average, but this effect did not exceed the bootstrap variation. For the percentage of people being exposed to high levels of air pollution and the percentage of urban population, there was no significant median effect. However, the bootstraps samples for the latter covariate show very high variation in the extremes (very high or very low percentage), compared to the median ranges. This might indicate differences in the average growth rate within the countries with very high or low percentage of urban population.
