# Supplementary material for: Onset of effects of non-pharmaceutical interventions on COVID-19 infection rates in 176 countries
Source: BMC Public Health. 2021 Jul 28;21:1472. doi: 10.1186/s12889-021-11530-0 (PMC8318058; doi:10.1186/s12889-021-11530-0)
Supplement: Supplementary file 2 — Additional file 2. Extended Methods. [file 12889_2021_11530_MOESM2_ESM.docx]

**Additional file 1: Extended Methods**

Data sources

Three data sources were used in this study. The first dataset consisted of the time series of cumulative confirmed COVID-19 cases [1], collected by the Johns Hopkins University Center for Systems Science and Engineering (JHU CCSE). It is compiled from various data sources, including the WHO, and it features daily data of the total number of confirmed cases in 214 countries. The numbers are generally reported on country level, but for some large countries (Australia, Canada, and China), cases are reported by state.

The second dataset is the COVID-19 government response event dataset (CoronaNet) [2]. It is a hand-coded collection of policy announcements regarding non-pharmaceutical interventions (NPIs) of 198 countries. The data collection is performed by a large international team of researchers and includes secondary review. The policies are categorised into multiple dimensions, including 16 different types (e.g., closure and regulation of schools, restriction and regulation of businesses, social distancing), the level of government initiating the action (national, provincial, municipal), the enforcement of the policy (mandatory or voluntary), as well as special dimensions for specific types like the directionality (inbound, outbound, or both) for external border restrictions. These policies and their categorisation will be used to derive NPIs to use in the analysis (see below).

For each individual policy, the date of its implementation in the respective country is recorded, along with a free-text describing the event. The dataset lists about 31500 individual policies.

The third dataset used was the World Bank’s dataset of development indicators [3]. In order to account for some variability between countries, we included four country-level development indicators in our analysis. In a simulation study, the difference in the effects of containment strategies was found to be most pronounced for the age group of 60+ versus the other age groups [4]. Hence, we included the percentage of population with ages of 65 years or more [5], which is the indicator that comes closest in terms of age groups. Spread of COVID-19 was found to follow different patterns in urban and in rural areas [6], hence we included the percentage of people living in urban territories (calculated from total population [7] and urban population [8]). Spread of airborne viruses has also been linked to atmospheric pollution like fine particulate matter (PM_2.5_) in measels [9], and more recently also to COVID-19 [10]. Hence, we also used the percentage of people that are exposed to PM_2.5_ air pollution values exceeding the WHO guideline value [11]. Another factor that might influence the growth rate is the relative wealth of the inhabitants. For example, poverty has been linked to reduced compliance to shelter-in-place protocols [12]. As a proxy, we have included the Gross Domestic Product per capita at purchasing power parity (GDP/PPP) [13]. Where available, we have downloaded the data for the last full calendar year (2019), but for countries that did not provide current data, we used the most recent data of up to 10 years prior.

Calculating growth rates

For the analysis, the state-level information in the time series of cumulative confirmed COVID-19 cases was aggregated to country level (for Australia, Canada and China). The daily increase rate was calculated as the ratio of cumulative cases from one day to the next, for each country. To account for artefacts from variable reporting delays [14], we calculated a right-centred seven-day moving (geometric) mean, smoothing out weekend effects. This metric, the smoothed daily increase rate of cumulative confirmed COVID-19 cases, will be the main outcome metric for the analysis to follow. For simplicity, we will refer to this metric as the growth rate. A growth rate of one corresponds to zero new cases, i.e., no increase in cumulative cases from the previous day to the current day (within one country). A growth rate of 1.1 corresponds to a ten-percent increase.

The onset of new infectious diseases like COVID-19 is assumed to follow exponential growth [15–17], hence this ratio is expected to be constant over time if no NPIs are in place. Also, as the growth rate is calculated within countries, systematic over- or underreporting in a specific country (e.g., due to different testing strategies) should be effectively mitigated.

We only considered days that indicated 25 or more cumulative confirmed COVID-19 cases to calculate the smoothed growth rate in order to get more stable estimates, because the confirmed cases reported in the beginning of the epidemic show considerable variation (e.g., laboratory-confirmed results may take up to one week after testing until they are reported [e.g., 18, 19]). Additionally, we only used data from countries that provided at least 28 days of data. We only included countries in our analysis that the CoronaNet dataset of NPIs included data for. For a full list of countries used in the analysis, see Supplementary Table 2.

One of the assumptions of this study is that effectiveness of an NPI can be deduced from the reduction of the growth rate. This assumption is only valid in the beginning of the outbreak, as exponential growth is slowed in later phases by the implementation of NPIs and various other factors like changes in behaviour of the population in response to a global pandemic or saturation [20]. To model only the beginning, we limit our data to the first 90 days after the day that 25 cases have been reached for each country.

Because some countries changed reporting strategies in the course of the outbreak, the daily number of confirmed cases contains outliers. The most widely-known example is the day that China decided to include clinically diagnosed COVID-19 cases, alongside with laboratory tests (February 12, 2020), which led to 14108 new cases in a single day [21]. To mitigate the effects of such outliers, the growth rate was winsorised at one for the lower bound, as well as at the 99% quantile for the upper bound: all values larger than 1.335 have been set to this value.

Non-pharmaceutical interventions

For our analysis, we combined the individual policies listed in the CoronaNet dataset into NPIs by grouping them according to their type, the initiating country level (coded as national or sub-national), and the enforcement level (coded as mandatory, voluntary, or missing). For external border restrictions, we additionally grouped by the target direction (coded as inbound, outbound, both, or missing), as this category contained restrictions of citizens to travel abroad (outbound), as well as limitations for travellers to enter a specific country (inbound).

For each resulting NPI, the start date of the first policy in this group in each country was selected as the date of implementation (policies that did not list an implementation date were not considered). We excluded NPIs that were used in less than 20 countries, in order to get more stable and more generalisable estimates for the effects of the NPIs. This resulted in 57 NPIs, 35 of which were set on national level, and 22 on sub-national level. Regarding the enforcement level, 39 NPIs were mandatory, 18 were voluntary (1 NPI did not report any enforcement level). For a list of all NPIs used in the analysis, see Supplementary Table 1.

Combining the datasets and calculating features

The time series of cumulative confirmed COVID-19 cases was enriched with the country-level indicators from the world bank data, providing static covariates for each country. This should enable the machine learning model to estimate country-specific variations in growth rate, if they are related to the covariates. For the minority of countries in our sample that did not provide data (6.3% for the percentage of people being 65 years or older, 1.3% for the percentage of people living in urban territories, 5.1% the percentage of people that are exposed to air pollution, and 8.9% for the GDP per capita), missing values in the world bank indicators were imputed with the median value. GDP per capita was log-transformed because of high skewness in the data.

For each NPI, a new feature was derived based on the date of first implementation (start date of the first policy in this category) and its relation to the current date in the time series of confirmed cases in the respective country. Each of those features was calculated by taking the difference of the current date in the time series and the point in time each NPI was first implemented in the respective country. This number is zero on the day an NPI was implemented, and a positive integer after the NPI came into effect, representing the number of days that an NPI has been in place. This allows to investigate the effect of an NPI depending on how long it has been in place. To also investigate the trend in the COVID-19 growth rate before the NPI was implemented, the feature was allowed to be negative, encoding the number of days before the NPI came into effect. We capped the value at -14, with all smaller (earlier) values being set to this value, hence capturing the effect also during two weeks prior to NPI implementation. For countries that did not implement a specific NPI, the feature variable was set to a value of -15, allowing the machine learning model to distinguish between countries that have put an NPI in place and those that have not. Using a feature coding like this allows a non-linear machine learning model to estimate the strength of an NPI on each individual day. Starting from 14 days prior to implementation additionally allows to assess the validity of estimated model effects (effects should only occur after implementation).

In addition, two features that capture NPI-independent, time-related changes in the growth rate have been used in the analysis. First, an absolute time scale measuring the days in relation to 11 March 2020, the day that COVID-19 has been declared a pandemic by the WHO. This should allow for capturing differences in growth rates for countries that have faced the outbreak earlier (like China where the outbreak started) or later (like African countries) during the worldwide outbreak. The second feature is a relative time scale within each country. It measures the time relative to the day that 25 cases have been reached. This should allow to capture changes in growth rates that are unrelated to NPIs but depend on the timing of the local outbreak like increasing awareness of the danger [22, 23] and related changes in behaviour that might affect the growth rate.

The final dataset for analysis included 57 NPIs, two time-related covariates that are independent from the NPIs, as well as four country-specific covariates. The dataset contains the daily growth rate for 14559 day/country-combinations from 176 countries, up to 17 August 2020.

Fitting the machine learning model

To analyse the effect of the different NPIs, we fitted a random forest regression model [24]. The dependent variable was the growth rate, and the features consisted of four country-specific covariates, two time-related covariates, and 57 NPIs, each representing how long a certain NPI has been in place or if it has been in place at all (as described above). Note that we do not treat this data as time series data in our analysis. Due to the exponential growth in cumulative COVID-19 cases, the growth rate is expected to be constant at a certain value if no NPIs are in place, and independent of the day before or the day after. The actual value should only depend on the set of NPIs that currently are in place in each country on a given day. Neither the actual date nor the country that the daily value of the growth rate originally stems from are used as features in the model.

The data was split into training and test set using a 60%/40% split blocked by country, i.e., the data of a specific country was either completely in the training or in the test set (and not spread across both sets). This allows the model to be validated on data from the roughly 40% of the countries that it has not been trained on. The training set was used for hyperparameter tuning. We used a random search as hyper-parameter optimization strategy [25], performing 100 iterations with ten-fold cross-validation within the training set. To reduce overfitting, we used a restricted hyperparameter search space. The best resulting model was refit on the complete training set, and performance was estimated on the test set.

To estimate uncertainty in the model predictions as well as in the estimated effects of the NPIs, we used bootstrapping. From the training set, we drew 100 bootstrap samples, also blocked by country, i.e., randomly choosing countries (all days of the respective country) until the size of the data set matched the original training set. We then refit the model on these bootstrap samples (without hyperparameter tuning).

Even though we fit a model to predict the COVID-19 growth rates in different countries, this is not the focus of the study. Fitting this model is merely a means to an end. We are interested in understanding how and when different NPIs affect the growth rate, and the methodology to achieve this is described in the next subsection. However, this methodology is based on the machine learning model, hence we report the fit statistics here in the extended Methods section.

The model fit on the complete training set, as well as the bootstrap models, were able to approximate the data reasonably well, given we can only estimate an average effect over all countries. Fit statistics (Supplementary Table 3) indicate a slight overfit in the training set, but the models were able to explain about 47% of variance in the test set (on average in the bootstrap samples). Additionally, we used the models to predict the time series of growth rates for each individual country in our dataset, given the sequence of NPIs that have been chosen by that country. Supplementary Fig. 6 shows examples of reasonable fits of the models. It can clearly be seen that the models predict average effects of the NPIs in place, i.e., they have learned the average effect of the NPIs (see Results), which works well for some countries, but not so well for others (Supplementary Fig. 7). Common mistakes the models make are that they cannot predict untypically high growth rates or a resurgence of the growth rate after an initial decline (which might occur, for example, due to increased testing or because some NPIs or policies are no longer in place, e.g., if they do not have a reported end date in the CoronaNet dataset). The onset of the reduction of the growth rate is predicted slightly too early or too late in some cases, and the models cannot predict time series with untypical trends in the growth rate (Supplementary Fig. 7).

Assessing effects of NPIs

To understand how each NPI influenced the growth rate (according to the random forest regression model), accumulated local effect (ALE) plots [26] were used. These plots show what the model has learned about the influence of a specific feature over the whole value range of that feature. In our case, because of the way the features were designed, they show how the growth rate changes in relation to a specific NPI, from two weeks prior to implementation to 60 days after. They show the main effect of a feature at a certain value, compared to the average prediction, and they are centred at zero. Compared to similar plots like partial dependence plots, ALE plots do not suffer from the extrapolation problem, are not biased by the omitted variable phenomenon, and are well-suited to handle correlated features [27]. ALE plots do not provide a means to assess uncertainty, but they are much faster to calculate compared to their alternatives. This allows to use bootstrap sampling to assess uncertainty. The plots show what the model has learned and do not depend on the dataset that is used to draw the plots (except for random fluctuation in the feature range). Hence, we have bootstrapped the model training process (as described above) and we show ALE plots of the bootstrapped models to allow assessment of uncertainty. The plots are constructed using the full dataset and show the individual bootstrap results, the median effect over all bootstraps, as well as the model which has been estimated on the complete training set.

**References**

1. Novel Coronavirus (COVID-19) Cases Data - Humanitarian Data Exchange. https://data.humdata.org/dataset/novel-coronavirus-2019-ncov-cases. Accessed 23 Apr 2020.

2. Cheng C, Barceló J, Hartnett AS, Kubinec R, Messerschmidt L. COVID-19 Government Response Event Dataset (CoronaNet v.1.0). Nat Hum Behav. 2020;4:756–68.

3. Piburn J. wbstats: Programmatic Access to the World Bank API. Oak Ridge, Tennessee: Oak Ridge National Laboratory; 2018. https://www.ornl.gov/division/csed/gist.

4. Wilder B, Charpignon M, Killian JA, Ou H-C, Mate A, Jabbari S, et al. Modeling Between-Population Variation in COVID-19 Dynamics in Hubei, Lombardy, and New York City. SSRN Scholarly Paper. Rochester, NY: Social Science Research Network; 2020. doi:10.2139/ssrn.3564800.

5. Population ages 65 and above (% of total population) | Data. https://data.worldbank.org/indicator/SP.POP.65UP.TO.ZS. Accessed 28 Jul 2020.

6. Rao ASRS, Vazquez JA. Identification of COVID-19 can be quicker through artificial intelligence framework using a mobile phone-based survey in the populations when cities/towns are under quarantine. Infect Control Hosp Epidemiol. 2020. doi:10.1017/ice.2020.61.

7. Population, total | Data. https://data.worldbank.org/indicator/SP.POP.TOTL. Accessed 28 Jul 2020.

8. Urban population | Data. https://data.worldbank.org/indicator/SP.URB.TOTL. Accessed 28 Jul 2020.

9. Peng L, Zhao X, Tao Y, Mi S, Huang J, Zhang Q. The effects of air pollution and meteorological factors on measles cases in Lanzhou, China. Environ Sci Pollut Res. 2020;27:13524–33.

10. Fattorini D, Regoli F. Role of the chronic air pollution levels in the Covid-19 outbreak risk in Italy. Environ Pollut. 2020;264:114732.

11. PM2.5 air pollution, population exposed to levels exceeding WHO guideline value (% of total) | Data. https://data.worldbank.org/indicator/EN.ATM.PM25.MC.ZS. Accessed 28 Jul 2020.

12. Wright AL, Sonin K, Driscoll J, Wilson J. Poverty and Economic Dislocation Reduce Compliance with COVID-19 Shelter-in-Place Protocols. SSRN Scholarly Paper. Rochester, NY: Social Science Research Network; 2020. doi:10.2139/ssrn.3573637.

13. GDP per capita, PPP (current international $) | Data. https://data.worldbank.org/indicator/NY.GDP.PCAP.PP.CD?end=2019&start=1990. Accessed 28 Jul 2020.

14. WHO. Coronavirus disease 2019 (COVID-19) situation report 97. 2020. https://www.who.int/docs/default-source/coronaviruse/situation-reports/20200426-sitrep-97-covid-19.pdf?sfvrsn=d1c3e800_6. Accessed 26 Apr 2020.

15. Jung S, Akhmetzhanov AR, Hayashi K, Linton NM, Yang Y, Yuan B, et al. Real-time estimation of the risk of death from novel coronavirus (COVID-19) infection: inference using exported cases. J Clin Med. 2020;9:523.

16. Ma J. Estimating epidemic exponential growth rate and basic reproduction number. Infect Dis Model. 2020;5:129–41.

17. Muniz-Rodriguez K, Chowell G, Cheung C-H, Jia D, Lai P-Y, Lee Y, et al. Doubling Time of the COVID-19 Epidemic by Chinese Province. medRxiv. 2020. doi:10.1101/2020.02.05.20020750.

18. WHO. Coronavirus disease 2019 (COVID-19) situation report 12 (Indonesia). 2020. https://www.who.int/docs/default-source/searo/indonesia/covid19/who-situation-report-12.pdf?sfvrsn=811c7f19_2. Accessed 28 Jul 2020.

19. WHO. Coronavirus disease 2019 (COVID-19) situation report 183. 2020. https://www.who.int/docs/default-source/wha-70-and-phe/20200721-covid-19-sitrep-183.pdf?sfvrsn=b3869b3_2. Accessed 28 Jul 2020.

20. Singer HM. The COVID-19 pandemic: growth patterns, power law scaling, and saturation. Phys Biol. 2020. doi:10.1088/1478-3975/ab9bf5.

21. Brouwer ED, Raimondi D, Moreau Y. Modeling the COVID-19 outbreaks and the effectiveness of the containment measures adopted across countries. medRxiv. 2020. doi:10.1101/2020.04.02.20046375.

22. Harper CA, Satchell LP, Fido D, Latzman RD. Functional Fear Predicts Public Health Compliance in the COVID-19 Pandemic. Int J Ment Health Addict. 2020;:1–14.

23. Dryhurst S, Schneider CR, Kerr J, Freeman ALJ, Recchia G, Bles AM van der, et al. Risk perceptions of COVID-19 around the world. J Risk Res. 2020;0:1–13.

24. Wright MN, Ziegler A. ranger : A Fast Implementation of Random Forests for High Dimensional Data in C++ and R. J Stat Softw. 2017;77. doi:10.18637/jss.v077.i01.

25. Bergstra J, Bengio Y. Random Search for Hyper-Parameter Optimization. J Mach Learn Res. 2012;13:281–305.

26. Apley DW, Zhu J. Visualizing the effects of predictor variables in black box supervised learning models. ArXiv161208468 Stat. 2019. http://arxiv.org/abs/1612.08468. Accessed 20 Apr 2020.

27. Molnar C. Interpretable Machine Learning. https://christophm.github.io/interpretable-ml-book/. Accessed 20 Apr 2020.
